# Planetare Gesundheit und psychische Gesundheit

**DOI:** 10.1007/s00115-024-01742-1

**Published:** 2024-10-17

**Authors:** Charlotte M. Grosskopf, Helen Dauterstedt, W. Emanuel Severus, Michael Bauer, Klaus Michael Reininger, Katharina Scharping, Christoph Nikendei

**Affiliations:** 1 Zentrum für Seelische Gesundheit, Klinik und Poliklinik für Psychiatrie und Psychotherapie, Universitätsklinikum Carl Gustav Carus Dresden an der Technischen Universität Dresden, Fetscherstraße 74, 01307 Dresden, Deutschland; 2 RPK Gut Gamig, Dresden, Deutschland; 3 Klinik für Persönlichkeits- und Traumafolgestörungen, Asklepios Klinik Nord – Ochsenzoll, Hamburg, Deutschland; 4 Institut für Psychotherapie, Universitätsklinikum Hamburg-Eppendorf, Hamburg, Deutschland; 5 Klinik und Poliklinik für Psychosomatische Medizin und Psychotherapie, Universitätsklinikum Hamburg-Eppendorf, Hamburg, Deutschland; 6 Fachkrankenhaus für Psychiatrie und Psychotherapie, Neurologie und Psychosomatik, Dr. von Ehrenwall’sche Klinik, Ahrweiler, Deutschland; 7 Klinik für Allgemeine Innere Medizin und Psychosomatik, Universitätsklinikum Heidelberg, Heidelberg, Deutschland

**Keywords:** Klimawandel, Klimakrise, Globale Gesundheit, Gesundheitsbedrohung, Canmore-Erklärung, Climate change, Climate crisis, Global health, Threat to health, Canmore Declaration

## Abstract

Der Klimawandel stellt die größte globale Gesundheitsbedrohung des 21. Jahrhunderts dar. Treibhausgasemissionen und die hierdurch steigenden Durchschnittstemperaturen führen zu Hitzewellen, Dürre, Wasser- und Nahrungsmangel, Extremwetterereignissen, dem Anstieg des Meeresspiegels, Migrationsbewegungen sowie dem Verlust von Biodiversität und dem Untergang von Ökosystemen, wie wir sie kennen. Bereits dafür, dass die globale Durchschnittstemperatur bis 2029 maximal um 1,5 °C steigt, sind nach aktuellen Schätzungen viel umfangreichere gesellschaftliche Maßnahmen als bisher erforderlich. Doch nicht nur der Klimawandel, sondern auch andere menschengemachte Faktoren wie Lärm, Licht, Feinstaub und Plastik bedrohen die Gesundheit unseres Planeten und damit unweigerlich auch die Gesundheit des Menschen – sowohl die körperliche als auch die psychische. Dieser Artikel erweitert das DGPPN-Positionspapier „Klimawandel und psychische Gesundheit“ um den Begriff Planetare Gesundheit. Insbesondere die normative Dimension der Canmore-Erklärung zur Planetaren Gesundheit, Transdisziplinarität als Wissenschaftsprinzip sowie mögliche konkrete Handlungsanweisungen sollen hier in ihrer besonderen Relevanz für die Psychiatrie, Psychotherapie und die sprechende Medizin dargestellt und die Zusammenhänge grafisch illustriert werden.

## Abstract (en)

Climate change is the greatest global health threat of the twenty-first century. Greenhouse gas emissions and the resulting rise in average temperatures are directly linked to heatwaves, droughts, water and food shortages, extreme weather events, rising sea levels and migration movements as well as the loss of biodiversity and the alteration and degradation of ecosystems as we know them. Current estimates suggest that societies need to show much greater efforts to ensure that the global average temperature does not rise by more than 1.5 °C by 2029. Not only climate change but also other man-made factors, such as noise, light, particulate matter and plastics are threatening the health of the planet and therefore inevitably human health as well, both physical and mental health. Poorer planetary health also has an impact on human mental health. This article extends the DGPPN 2023 position paper on climate change and mental health to include the concept of planetary health. In particular, the normative dimension of the Canmore Declaration on Planetary Health, the concept of transdisciplinarity and specific calls for action are presented with their relevance to psychiatry, psychotherapy and mental health and the associations are graphically illustrated.

## Planetare Gesundheit und das Anthropozän

Das Konzept der Erde als Patientin, die durch die Ausbeutung ihrer Ressourcen durch uns Menschen krank wurde und nun ärztlicher und psychotherapeutischer Hilfe bedarf, wurde bereits 1994 von Per Fugelli in seinem Artikel „In Search of a Global Social Medicine“ vorgestellt [17], in dem er auf die weitreichenden Gefahren hinwies, die durch die Erkrankung der Erde auch für die menschliche Gesundheit resultieren könnten. Der Begriff Planetare Gesundheit (Planetary Health) wurde schließlich 2015 in dem Bericht der „Rockefeller Foundation-Lancet“-Kommission einem breiteren Publikum vorgestellt, und definiert als die „Gesundheit der menschlichen Zivilisation und der natürlichen Systeme, von welchen sie abhängt“ [32, 41]. Weitreichende Veränderungen der natürlichen Systeme der Erde durch eine nicht nachhaltige Nutzung derselben stellen eine wachsende Bedrohung für die menschliche Gesundheit und die gesamte menschliche Zivilisation dar. Erwähnt werden in diesem Kontext u. a. der Klimawandel, die Übersäuerung der Ozeane [22], die veränderte Landnutzung [16, 22, 36], Landverödung, Wasserknappheit [22, 36], Überfischung, Eutrophierung (d. h. Nährstoffanreicherung durch Stickstoffdünger und folglich verursachtes Bakterien- und Algenwachstum), (Land‑, Luft- und Meeres‑)Verschmutzung und ein Verlust der biologischen Vielfalt. Dieser Artikel möchte die Konzepte „Planetare Gesundheit“, „Anthropozän“ sowie „Transdisziplinarität“ erläutern und miteinander in Verbindung setzen, um damit das Positionspapier der Taskforce der Deutschen Gesellschaft für Psychiatrie und Psychotherapie, Psychosomatik und Nervenheilkunde (DGPPN) [20] zu erweitern. Gleichzeitig wollen wir eine transdisziplinäre Einordnung des bisherigen Wissensstandes und die Relevanz für die Psychiatrie herausstellen und konkrete Handlungsempfehlungen (s. das DGPPN-Positionspapier erreichbar über den QR-Code in Infobox 3) aufzeigen.

Der Begriff des Anthropozäns wird in diesem Zusammenhang für die Kennzeichnung einer neuen geologischen Epoche verwendet, in der unsere menschlichen Aktivitäten begannen, einen erheblichen globalen Effekt auf die Systeme der Erde auszuüben, deren planetare Belastbarkeitsgrenzen auszuloten und zu überschreiten [34]. Als ein möglicher Beginn wird das späte 18. Jahrhundert diskutiert, als die globale Konzentration der für den Klimawandel wesentlich verantwortlichen Treibhausgase CO_2_ sowie Methan und Lachgas anzusteigen begann [12]. Von forschenden Experten zur Quartärstratigraphie (Teilbereich der Geologie, Arbeitsgruppe der „International Union of Geological Sciences“) wurde eine Einführung des Begriffs „Anthropozän“, und damit eine Ablösung des „Holozäns“ als geologische Erdepoche, im März 2024 abgelehnt [42]. Unter anderem wurde argumentiert, dass das Anthropozän als „Ereignis“ in der geologischen Geschichte definiert werden sollte, aber nicht als eine formale Epoche. Außerhalb der Stratigraphie und im Kontext Planetarer Gesundheit stellt der Begriff des Anthropozäns jedoch (weiterhin) einen wichtigen Begriff dar, der die gegenwärtige Situation der Erde, in Abgrenzung zu früheren Zeitpunkten, aus Sicht der heute hier lebenden Menschen beschreibt [34, 42]. Die Etablierung und Nutzung des Begriffs „Anthropozän“ kann hier als Beispiel für Transdisziplinarität bzw. transdisziplinäre Begriffsarbeit dienen. Transdisziplinarität wurde u. a. vom Wissenschaftsphilosophen Jürgen Mittelstraß 2005 definiert: „Transdisziplinarität wird als ein Forschungs- und Wissenschaftsprinzip verstanden, das überall dort wirksam wird, wo eine allein fachliche oder disziplinäre Definition von Problemlagen und Problemlösungen nicht möglich ist bzw. über derartige Definitionen hinausgeführt wird“ [25]. Es stellt sich hierbei nicht die Frage, ob die mess- und datierbaren Veränderungen dem geowissenschaftlichen Konsens zur Nomenklatur von Erdzeitaltern genügen, sondern ob eine Beschreibung der komplexen und vielfachen Veränderungen für die heute lebenden Menschen und deren Systeme unter einem Begriff näherungsweise zusammengefasst werden kann. Der Begriff Anthropozän wird daher im Kontext Planetarer Gesundheit weiterhin im Sinne eines kulturellen Konzepts benutzt, um die von Menschen verursachte Veränderung planetarer Lebensbedingungen darzustellen.

Seit dem ersten *Lancet*-Bericht im Jahr 2015 hat sich der Zustand unseres Planeten Erde in vielerlei Hinsicht weiter deutlich verschlechtert. Im Vordergrund stehen hierbei der Klimawandel und der Verlust der Biodiversität. Hinsichtlich des ersten Punkts kommen UN-Berichte zu dramatischen Ergebnissen [22, 23]. So müssten die CO_2_-Emissionen bis 2030 um etwa die Hälfte sinken, um das international vereinbarte Ziel von maximal 1,5 °C Erderwärmung zu erreichen. Diese steigen weltweit jedoch nach wie vor weiter an, und im Bereich Klimaforschung werden vermehrt Stimmen laut, die anhand der Messungen der letzten Jahre eine noch höhere Klimasensitivität unseres Planeten für wahrscheinlich halten [18]. Die Klimasensitivität ist eine Variable in Klimamodellrechnungen, die beschreibt, welcher globale Temperaturanstieg bei einem Anstieg (insbesondere: Verdopplung) der Menge freigesetzten Treibhausgases CO_2_ zu erwarten ist. Demnach würde im Rahmen der bisherigen Emissionen und Regulationen der globale Durchschnittstemperaturanstieg bereits in den 2020er-Jahren 1,5 °C und vor 2050 2 °C überschreiten [18]. Die bisherigen Berichte des Weltklimarats gingen noch von einer etwas geringeren Klimasensitivität aus, aktuell wird gemäß der bis 2030 gemeldeten Daten eine Erwärmung um +2,8 °C (+2,1 °C bis +3,4 °C) bis 2100 erwartet, wenn die politischen Maßnahmen nicht massiv gesteigert werden [23, S. 11].

**Abb. 1:**
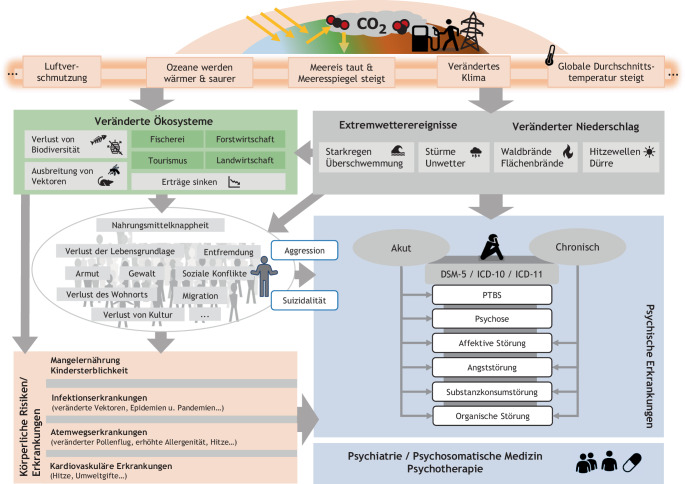
Schematische Darstellung einiger bekannter Zusammenhänge zwischen anthropogener Umweltveränderung und psychischer Gesundheit. (Adaptiert nach [22, 10, 38])

Hinsichtlich des Verlustes der biologischen Vielfalt deutet vieles darauf hin, dass wir uns inmitten eines großen Artensterbens befinden [8, 33]. Zusätzlich bedroht eine von Menschen beeinflusste Umwelt auch durch Lärm, Licht, Feinstaub, (Psycho‑)Pharmaka und Plastik die menschliche Gesundheit, im Sinne einer „triple planetary crisis“ („climate change“, „pollution“ and „biodiversity loss“; [2, 24, 28]). All diese Faktoren, welche nur eine Auswahl darstellen, interagieren in mannigfaltiger Art und Weise miteinander und führen auch zu psychischen Erkrankungen (Abb. 1 sowie DGPPN-Positionspapier).

Die insbesondere in der rechten Bildhälfte in Abb. 1 gezeigten Zusammenhänge zwischen Klimaveränderungen und psychischer Erkrankung sind wissenschaftlich belegt und werden u. a. im Positionspapier der DGPPN-Taskforce „Klima und Psyche“ erläutert [20].

## Klimawandel, (PSY-)Fachgesellschaften und Gesellschaft

Wie im DGPPN-Positionspapier umfangreich und anschaulich dargestellt, hat der Klimawandel und das Ziel der Klimaneutralität auch größte Relevanz für den Fachbereich Psychiatrie und Psychotherapie [20]. Die DGPPN veröffentlichte bereits 2019 eine Stellungnahme zu den Auswirkungen der Klimaveränderungen auf die psychische Gesundheit und war hierbei die erste deutsche Fachgesellschaft, die sich zum Klimawandel positionierte [44] und 2022 eine Taskforce berief, deren Ausarbeitungen 2023 in Form eines Positionspapiers [20] veröffentlicht und beim letzten DGPPN-Kongress 2023 im Rahmen verschiedener Symposien diskutiert wurden. Ebenso wurde 2021 auf dem Deutschen Psychosomatikkongress die AG „Klimakrise und Planetare Gesundheit“ der Fachgesellschaften Deutsche Gesellschaft für Psychosomatische Medizin und Ärztliche Psychotherapie (DGPM), Deutsches Kollegium für Psychosomatische Medizin (DKPM) und des Berufsverbands der Fachärztinnen und Fachärzte für Psychosomatische Medizin und Psychotherapie Deutschlands (BPM) gegründet, welche regelmäßige unterjährige Treffen, Newsletter und Symposien verantwortet. Weiterhin positionierten sich 2023 die Bundespsychotherapeutenkammer, die Deutsche Gesellschaft für Psychologie und weitere Vereinigungen. Auf europäischer Ebene regte die European Association of Psychosomatic Medicine ebensolche Initiativen an und die European Psychiatric Association veröffentlichte im Mai 2024 ein Positionspapier [7].

Für eine noch allgemeinere Einordnung ist die kürzlich, im März 2024, vom Deutschen Ethikrat veröffentlichte Stellungnahme zu Klimagerechtigkeit hervorzuheben, in welcher auch das Gesundheitssystem auf individueller, jedoch auch kollektiver Ebene angesprochen wird: „Angesichts der auch in Deutschland bereits jetzt schon erkennbaren und erwartet zunehmenden vielfältigen gesundheitlichen Folgen des Klimawandels trägt der Gesundheitssektor eine besondere Verantwortung, auf diese Herausforderungen zu reagieren und Schutzmaßnahmen umzusetzen“ (S. 107 [14]).

Jahrzehntelang war der Einfluss des Klimawandels nicht in der medizinischen Ausbildung enthalten; im aktuellen Nationalen Kompetenzbasierten Lernzielkatalog Medizin (NKLM) werden inzwischen Zusammenhänge wie „Solastalgie als neuer Risikofaktor für Depression“ im Kompetenzbereich „Affektive Erkrankungen“ als Fokuswissen mit Ziel der Handlungskompetenz geführt (siehe https://nklm.de/). Neben der Anpassung von Lehrinhalten erfordert dies ein Aneignen der Wissensinhalte im Rahmen von Fort- und Weiterbildung. Gleichzeitig zeigt der bisherige Wissensstand, transdisziplinär eingeordnet, dass der Umgang mit diesem Wissen und den Veränderungen Anstrengung und Kompetenzen erfordert und eine besonders emotional herausfordernde Tätigkeit darstellt [19, 26, 30]. Die Mitinbegriffenheit klinisch-psychiatrisch tätiger Menschen in der Klimakrise begründet, dass „eine allein fachliche oder disziplinäre Definition von Problemlagen und Problemlösungen nicht möglich ist bzw. über derartige Definitionen hinausgeführt wird“, was nach Mittelstraß Transdisziplinarität charakterisiert (siehe Infobox 1).

## Der Begriff „Planetare Gesundheit“ umfasst mehr

Planetare Gesundheit versucht, die komplexen und disziplin- und sektorübergreifenden Einflussfaktoren in ihrer wechselseitigen Abhängigkeit zu begreifen. Ein wissenschaftlicher Konsens verschiedener Experten wurde hierzu im April 2018 in der Canmore-Erklärung [32, 36] formuliert und erweitert dabei die Ottawa-Charta zur Gesundheitsförderung von 1986 der WHO [40]. Hierbei wird in Form von 10 Prinzipien explizit die dringende Notwendigkeit herausgestellt, die Gesundheitsbedingungen von Mensch und Planet als *untrennbar* miteinander verbunden zu betrachten. Planetare Gesundheit wird hier definiert als die „voneinander abhängige Vitalität aller natürlichen und anthropogenen (d. h. auch sozialen, politischen und wirtschaftlichen) Ökosysteme auf Mikro‑, Meso- und Makroebene“ [32]. Hierbei nehmen Einstellungen, Werte, Verhaltensweisen sowie Beziehungen eine zentrale Rolle beim Erreichen planetarer Gesundheitsziele ein. Die Vitalität der Ökosysteme ist reziprok abhängig von „Empathie, Freundlichkeit, Gegenseitigkeit, Verantwortungsübernahme und Austausch“ auf individueller, gemeinschaftlicher, gesellschaftlicher und globaler Ebene. Im Kontext personalisierter Medizin sollte, wo immer es möglich ist, das „Verständnis für unsere Abhängigkeit von der natürlichen Umwelt, die uns umgibt“ (Flora, Fauna und unsere physische Welt) und dem „intimen Teil von uns“ (dem „menschlichen Mikrobiom“) gefördert werden (biopsychosoziale Interdependenz) [32].

Die Planetare Gesundheit erfordert gemäß der Canmore-Erklärung „ein Gespür“ und eine Reflexion psychologischer Einflussfaktoren wie soziale Dominanz und Elitismus, um keine Stimmen zu marginalisieren und die erklärten Ziele der Weltgesundheitsorganisation für die globale Gesundheitsförderung nicht zu behindern. Ebenso sollte Forschung in allen Bereichen mit einer „sinnvollen Einbindung der Gemeinschaft“ und mit Partnerschaften erfolgen, die die Motivation und die begünstigten Personen der Forschungsagenda sorgfältig beleuchten. Handlungsbezogen werden die oben genannten Aspekte in der Canmore-Erklärung auch als ethische Verpflichtung zur Gestaltung neuer normativer Verhaltensweisen formuliert: „Wir sollten mit gutem Beispiel vorangehen und uns bemühen, im klinischen/akademischen/öffentlichen Umfeld und darüber hinaus einen Lebensstil zu führen, der sich an der Erhaltung der planetaren Gesundheit orientiert. Wir sollten uns für eine globale Friedensagenda einsetzen, Gegenseitigkeit, Empathie und Zusammenhalt in der Gemeinschaft fördern und unterstreichen, dass Aggression, Konflikte und Gewalt zerstörerisch für Menschen, Natur und Planeten sind“ [32].

Die Canmore-Erklärung stellt dar, dass für eine Annäherung an Klimaneutralität Veränderungen in verschiedenen Bereichen menschlicher Aktivität und zwischenmenschlicher Interaktion notwendig sind. Dies erfordert, je nach Bezugssituation, die Fähigkeit zum (globalen) Perspektivwechsel über das bisher bekannte Lebensumfeld hinaus. Andernfalls droht ein Ungleichgewicht zwischen der menschlichen Gesundheit, den politischen, ökonomischen und sozialen Systemen, so wie den natürlichen Systemen unseres Planeten. Resultierende Hitze, Trockenheit, Dürre, Extremwetterereignisse werden einerseits zu konkret fassbaren Belastungen und andererseits zu einer gesteigerten Wahrnehmung der Klimakrise und zu einem Mehr an „Klimagefühlen“ führen. Dieses Wissen (wie u. a. im NKLM gefordert) bildet sich in unterschiedlichen Themenbereichen ab. Für Praktizierende im Bereich Psychiatrie und Psychotherapie ist daher ein Verständnis emotionaler, diagnostischer sowie geografischer Aspekte für die Einbeziehung eines planetaren Bewusstseins in den Arbeitsalltag zentral:

## Klimagefühle

Im Rahmen der Bewusstwerdung der dramatischen Folgen des Klimawandels für das eigene Menschsein ist es naheliegend, dass Empfindungen wie Angst, Trauer, Überraschung, Enttäuschung, Wut, Hass, Scham, Ekel, Neid, Schuld und Schmerz aufkommen [19, 30]. Diese werden in diesem Kontext oft als „Klimagefühle“ bezeichnet und nicht per se als pathologisch, sondern im Sinne menschlichen Denkens und Empfindens als normale Reaktion eingeordnet [15, 31]. Hierbei können Basisemotionen, aber auch komplexe Gefühle (Ehrfurcht, Ratlosigkeit, Nostalgie …) auftreten. Je nach Kontext lassen sich nicht nur negative, sondern auch neutrale oder positive Gefühle (Freude, Liebe, Hoffnung, Wertschätzung, Zugehörigkeit …) bei klimapsychologischen Themen identifizieren. Die Wahrnehmung und der Umgang mit Klimagefühlen sind für die Entwicklung von Resilienz zentral. Sobald Klimagefühle ein Ausmaß erreichen, dass diese die Alltagsfunktion beeinträchtigen, ist jedoch von einer neuen psychischen Belastungsform zu sprechen.

## Neue psychische Belastungsformen

Weltweit ließ sich in den letzten 15 Jahren ein erstmaliges Auftreten und ein deutlicher Anstieg direkt mit der Klimakrise zusammenhängender psychischer Belastungsformen verzeichnen [20]. Begriffe wie „eco anxiety“ oder „climate anxiety“ ([13, 21], umwelt- und klimabezogene Ängste), „climate grief“ ([13], klimabezogene Trauer als Antwort auf das Begreifen der allumfassenden Zerstörung unserer Lebensvoraussetzungen) oder Solastalgie ([1], durch Umweltveränderung verursachtes physisches und psychisches Stresserleben) wurden hierfür vorgeschlagen [27].

Es wird weiterhin diskutiert, inwiefern dies als neue, auf das Individuum bezogene, Krankheitsentität aufzufassen ist oder eher als nicht krankhafte, sondern begründete Reaktion gedeutet werden sollte, die eher im Konflikt mit soziokulturellen Strukturen zu Beeinträchtigungen führt [13, 15].

Insbesondere in der Altersgruppe der Jugendlichen und jungen Erwachsenen zeigt sich eine erhöhte Prävalenz: In einer weltweiten Umfrage von Hickman et al., bei der insgesamt 10.000 16- bis 25-Jährige aus 10 Ländern befragt wurden, zeigte sich ein Großteil über den Klimawandel besorgt (59 % sehr oder extrem besorgt, 84 % mindestens mittelgradig besorgt; [21]). Mehr als 50 % der Befragten berichteten von den folgenden Gefühlen: traurig, ängstlich, wütend, machtlos, hilflos und schuldig.

## Regionale Unterschiede in der Alltagsbeeinträchtigung

Mehr als 45 % der befragten jungen Menschen gaben an, dass sich ihre Gefühle bezüglich der Klimakrise negativ auf ihr tägliches Leben und ihr Funktionieren auswirken. Am häufigsten wurden Alltagsbeeinträchtigungen in Indien und den Philippinen (je 74 %) und Nigeria (66 %) berichtet. In den nördlichen Ländern USA, Vereinigtes Königreich und Finnland waren deutlich weniger Befragte davon betroffen (USA 26 %, UK 28 %, Finnland 31 %).

Insgesamt gaben 75 % der im Jahr 2021 Befragten an, dass sie die Zukunft für beängstigend halten. 39 % äußerten, sich unsicher zu sein, ob sie Kinder haben wollten. 65 % gaben an, dass Regierungen junge Menschen auf der ganzen Welt im Stich lassen würden, und 55 % stimmten der Aussage zu, die Menschheit sei dem Untergang geweiht („humanity is doomed“), ebenso mit regionalen Unterschieden (74 % in Indien vs. 43 % in Finnland; [21]).

## Beschäftigung mit Klimathemen in der Wissenschaft belastet

Nicht nur im Privaten können diese Belastungsformen auftreten. Auch Klimaforschende leiden emotional unter der Beschäftigung mit den Erkenntnissen rund um Erderwärmung, Massenaussterben und Ressourcenknappheit [9].

„Als Reaktion auf […] soziale Normen, Mechanismen des Wissenschaftsbetriebs, […] Interaktion mit Klimaleugnern und zum Schutz des eigenen Ichs und der Familie setzen Klimaforschende eine Reihe von Verhaltensweisen und Strategien ein, um ihre Emotionen im Zusammenhang mit dem Klimawandel und der Zukunft zu steuern“ [übersetzt aus 19].

Hier gehöre jedoch auch die zweckorientierte Verleugnung oder Verdrängung dazu: „die Betonung der Leidenschaftslosigkeit, die Unterdrückung schmerzhafter Emotionen und der Einsatz von schwarzem Humor“.

Für die Zukunft verdeutlicht dies die Notwendigkeit der Anerkennung und achtsamen Integration dieser affektiven Begleitaspekte im Rahmen wissenschaftlicher Tätigkeit. Hieraus erklärt sich auch die Relevanz inter- und transdisziplinären Austausches, um insbesondere auf das Wissen und die Fertigkeiten der multidisziplinären Psychotherapie zugreifen zu können [39].

## Besondere Rolle der sprechenden Medizin

Wie im Positionspapier der DGPPN zusammengefasst, beinhaltet ein adäquater Umgang mit dem Klimawandel und dessen Folgen für die moderne Medizin sowie Psychiatrie und Psychotherapie insbesondere auch die Adaptation der klinischen Versorgung (Infrastruktur, Material und Abläufe sowie Behandlungsketten und -angebote) sowie auch des Fokus von Forschung und Wissenschaft sowie die Integration des Themas in die Aus- und Weiterbildung [20].

Die gesellschaftliche Rolle des Arztes bzw. des Psychotherapeuten bietet die Möglichkeiten einer Multiplikatorfunktion innerhalb einer Gesellschaft [4, 6, 26]. Gleichzeitig sind die Psychiatrie und die Psychotherapie an erster Stelle in Form von Mehrbelastung der Gesundheitsversorgung betroffen, wenn es um klimawandelbedingtes psychisches Leiden geht.

Als Psychiater und Psychotherapeuten werden wir uns in den nächsten Jahren noch häufiger mit sozialpsychiatrischen und umweltbezogenen Aspekten beschäftigen, als dies bereits jetzt der Fall ist. Wir werden zum einen mit der Gesellschaft, jedoch auch mit anderen medizinischen Disziplinen über perspektivisch deutlich knapper werdende Ressourcen, wie die Arbeitszeit von Fachkräften oder Gelder, verhandeln müssen (vgl. *Ärzteblatt*-Titel 04/2024: „Erste Bundesländer erreichen bald ‚Kipppunkt‘ beim Pflegepersonal“ [3]). Ebenso werden baulich-infrastrukturelle Aspekte der psychiatrischen Versorgung, nicht nur wie in der COVID-19-Pandemie geschehen, häufiger und mit mehr Brisanz, insbesondere bez. Hitzeschutz in Innenräumen, diskutiert werden. Eine bewusste Integration dieser Themen in das Berufsbild ist daher unvermeidbar und notwendig. Selbstfürsorge und achtsame Anerkennung eigener Grenzen, jedoch auch Nutzung der verfügbaren Handlungsspielräume sollten hier einbezogen werden.

Es ist aktuell auch davon auszugehen, dass noch existenziellere Grundlagen des Berufsbildes Arzt bzw. Psychotherapeut vor dem Hintergrund der Klimakatastrophe neu auszuloten sind. Die individuelle Haltung zu diesen Überlegungen kann unterschiedlich ausfallen; im disziplinären Diskurs zur Berufspraxis gehören sie jedoch zunehmend dazu und eine bewusste Auseinandersetzung mit diesen ist für die Perspektive auf Planetare Gesundheit (vgl. 10 Punkte, Infobox 2) zentral.

## Veränderung als Chance

Abschließend soll an dieser Stelle auch auf die „positiven Klimagefühle“ verwiesen werden. Die Bewusstwerdung über die gegenwärtige Situation in ihrer existenziellen Bedrohlichkeit und das Erkennen der problematischen Doppelrolle des Menschen als Verursacher und Leidtragender ist der notwendige erste Schritt, um bewusst weitere Schritte gehen zu können.

Im Gespräch mit Kollegen und im Freundeskreis können Informationen ausgetauscht, Sichtweisen vermittelt, Gefühle wahrgenommen und reflektiert und neue Handlungsalternativen entwickelt werden. Bisher errungene Teilerfolge, wie im individuellen Alltag gelungene Umsetzung von Handlungsempfehlungen oder eine wachsende Anerkennung der Thematik innerhalb der Psychiatrie (exemplarisch hier das Positionspapier der DGPPN), können ermutigen.

Planetare Gesundheit verbindet die Entwicklung neuer Lösungen mit dem rationalen Einsatz der bestehenden Möglichkeiten und Ressourcen. Die Erarbeitung von Neuem bezieht sich jedoch nicht primär auf heilsbringende Technologien – sondern vorrangig auf die Entwicklung neuer Narrative für ein menschliches Miteinander. Dass wir *alle* vom Planeten und dessen Ökosystemen abhängig sind, kann in Zeiten von Entfremdungs- und Isolationserleben durch unsere menschengemachte Welt auch Reorientierung, „Erdung“ und Mitmenschlichkeit bieten.

Ein auf das Anthropozän angepasster Gesundheitsbegriff, welcher die Biopsychosoziale Interdependenz und die real existierenden planetaren Grenzen reflektiert [34], steht hierbei insbesondere einem traditionellen Wachstumsdenken auch innerhalb der Medizin entgegen.

### Infobox 1 Transdisziplinäre Wissenschaftsreflexion

Wie ist nachgewiesen, dass Waldbrände und Überschwemmungen PTBS oder Depression verursachen? Woher stammt die Evidenz?

Zu Wald- und Flächenbränden gibt es schon seit vielen Jahren wissenschaftliche Studien (mind. 42 querschnittlich, mind. 20 Kohortenstudien, 1 RCT [37]), vor allem aus Australien, USA, Kanada und Griechenland (vgl. Review von 2021 [37]). Lassen sich dortige Erhebungen auf andere Länder, wie Deutschland, übertragen? Reicht das aus, um bei steigenden Waldbränden in Deutschland ebenso davon auszugehen, dass Menschen hier (Feuerwehrleute, Anwohnende etc.) ähnliche Symptome entwickeln? Müssten wir hierfür mehr eigene Studien erheben? Die Frage nach der Verallgemeinbarkeit konkreter Beobachtungen auf allgemeine Regeln, Theorien und Prinzipien wird im akademischen Diskurs häufig gestellt und entspricht wissenschaftstheoretisch dem Induktionsprinzip. Um zu allgemeingültigen Theoriewissen zu kommen, müssen relevante Einflussfaktoren identifiziert, gewichtet und allgemeine Zusammenhänge abstrahiert werden.

Welche kulturellen, politischen und ökonomischen Faktoren sind länderspezifisch zu berücksichtigen? Wie können diese erhoben werden? Welche Rolle für die in Deutschland anfallende „Krankheitslast“ durch psychische Folgen von Extremwetterereignissen spielt Migration? Zu all diesen Fragen gibt es bereits Forschungsansätze in Deutschland oder dem Ausland, welche jedoch oft nur innerhalb der jeweils eigenen Disziplin (Epidemiologie, Psychotraumatologie, Migrationsforschung u. ä.) wahrgenommen werden.

Die Taskforce „Klima und Psyche“ verwies bei der Sichtung der Datenlage und Erstellung des Positionspapiers insbesondere auf die Studien aus den USA zu Hurricane Katrina 2005. In Deutschland lässt sich anhand der Ahrtalflut vom 14.07.2021 die Dramatik der Problemlage und die praktischen Limitationen wissenschaftlicher Erhebung und Kommunikation zeigen:

**Im Rahmen der Flutkatastrophe wurde die Dr. von Ehrenwall’sche Klinik in Ahrweiler mit allen 13 Gebäuden vollständig zerstört.** Die Klinik konnte ihren Versorgungsauftrag über viele Monate nicht mehr erfüllen, akut behandlungsbedürftige Patienten mussten in benachbarten psychiatrischen Kliniken untergebracht werden. Erst im November 2021 konnten erste stationäre Patienten behelfsweise in einem Hotel behandelt werden. **Seit Oktober 2023, mehr als 2 Jahre später, wurde eine kleinere geschützte Station wieder in Betrieb und damit die Hälfte des ursprünglichen Versorgungsgebiets wieder übernommen.** Aktuell kann die Klinik 90 stationäre Behandlungsplätze anbieten, vor der Flut waren es 150 Krankenhaus- und 23 Rehabetten. Im Dezember 2021 eröffnete unter Leitung der Dr. von Ehrenwall’schen Klinik mit Förderung des Landes Rheinland-Pfalz ein **Traumahilfezentrum** mit einem niederschwelligen Beratungsangebot für Betroffene und Helfer. Insgesamt wurden dort seit der Eröffnung am 01.12.2021 bis zum 25.03.2024 3801 Einzelberatungen, 216 Gruppen und 63 Fortbildungen durchgeführt. Der Zulauf aus der Bevölkerung ist unverändert hoch. **Trotz Sonderzulassungen für Psychotherapeuten‑/Kinder-Jugend-Therapeuten ist der Therapiebedarf im Ahrtal noch nicht gedeckt, die Wartezeiten für einen Psychotherapieplatz betragen für Erwachsene und Kinder mehrere Monate.**

Am Beispiel der Ahrtalflut lassen sich die Auswirkungen des Extremwetterereignisses auf das Gesundheitssystem zeigen. Im Rahmen der Bewältigung einer schwierigen Versorgungssituation sowie weiterer Faktoren (u. a. kein vorbestehender Forschungsschwerpunkt, keine laufenden Forschungsprojekte) wird **greifbar, dass unter den genannten Umständen an der Dr. von Ehrenwall’schen Klinik bisher keine psychiatrisch-wissenschaftlichen Erhebungen durchgeführt wurden.** Fast alle bisherigen wissenschaftlichen Publikationen zur Ahrtalflut kommen aus dem Bereich Umweltwissenschaften, siehe **Publikationsliste Projektverbund KAHR** (BMBF-Projekt Klima-Anpassung, Hochwasser und Resilienz), siehe bspw. Wolf et al. [43], und beschäftigen sich primär mit Hochwasserschutz. Hier ließe sich transdisziplinär fragen: Wissen wir im psychiatrisch-psychotherapeutischen Kontext genug über die Behandlung der neuen Belastungsformen? Reicht umweltwissenschaftliche Forschung aus, oder bedarf es mehr klinisch-psychiatrischer und psychotraumatologischer Perspektiven, sowohl bei der wissenschaftlichen Analyse als auch bei der Handlungsplanung im politischen Diskurs?

## Ausblick und Fazit für die Praxis

Zusammenfassend gibt es umfangreiche Arbeiten zu den Auswirkungen des Klimawandels auf die menschliche Psyche [5, 10, 27, 29] und die menschliche Gesundheit [35, 38]. Der breite wissenschaftliche Konsens ist, dass sich eine Mehrbelastung historischen Ausmaßes erwarten lässt, was größte Auswirkungen für die öffentliche Gesundheit und insbesondere die Psychiatrie haben kann [6, 11, 26]. Die Themen klimawandelbedingte Umweltveränderungen und psychische Gesundheit sind empirisch stark miteinander verwoben (Abb. 1), weswegen die Sorge um Planetare Gesundheit auch die Sorge um die psychische Gesundheit von Patienten und Behandelnden bedeutet. Die Inhalte des DGPPN-Positionspapiers „Klimawandel und psychische Gesundheit“ müssen in der psychiatrischen, psychosomatischen und psychotherapeutischen Praxis mehr Beachtung, Rezeption und Anwendung finden.

Die Reflexion des Mitinbegriffenseins der eigenen Person in die natürlichen Systeme dieses Planeten (siehe Canmore-Erklärung) und ein Anerkennen biopsychosozialer Interdependenz bieten hier den Ausgangspunkt für eine individuelle Auseinandersetzung mit dem eigenen Handeln sowie für einen lösungsorientierten Diskurs auf interpersoneller Ebene und innerhalb unterschiedlicher Kollektive (private und nichtstaatliche Zusammenschlüsse).

### Infobox 2 Zehn zentrale Handlungsempfehlungen für eine klimaneutrale Psychiatrie (Anhang A1, Positionspapier DGPPN; [20])

1. Mental Health in all Policies: Mehr Prävention sowohl in der Psychiatrie als auch sektorübergreifend, z. B. durch Planung ausreichender und zugänglicher Grünflächen in Einrichtungen des psychiatrischen Hilfesystems, wie Zugang zu verschatteten Gärten mit Bäumen statt nur „Freiflächen“ bei Unterbringung nach PsychKG, Verringerung von Obdachlosigkeit und sozialer Isolation, Förderung von Beschäftigung bei Menschen mit psychischer Erkrankung.
2. Mehr Empowerment innerhalb des psychiatrischen Versorgungssystems (z. B. Förderung von Gesundheitskompetenz, Selbstsorge, Peer Support und Zugang zu Psychotherapie).
3. Integration der Thematik „Klima und Psyche“ in psychiatrischer Aus‑/Fort‑/Weiterbildung, Behandlung und Gestaltung der Hilfesysteme sowie Forschung (Erforschung der Folgen des Klimawandels und Entwicklung von Präventions- und Interventionsmaßnahmen).
4. Klinik- und Praxisinfrastruktur zur Energiewende nutzen (z. B. Installation von Photovoltaik, finanzierbar z. B. durch Contracting), Bezug von Ökostrom, Dämmung und Verschattung statt Klimaanlage (wo möglich), energetische Sanierung unter Beachtung von „grauer Energie“, d. h. Energie für Herstellung, Transport, Lagerung, Verkauf und Entsorgung.
5. Adaptation der Institutionen in der Psychiatrie an erwartbare Umweltveränderungen, z. B. Anpassung der Klinik- und Praxisinfrastruktur an Hitze, Anpassung an Zunahme von Depressionen, Angsterkrankungen und psychotischen Erkrankungen.
6. Vermeidung von Ressourcenverschwendung im Behandlungsablauf: Leitliniengerechte Optimierung des Medikamenten- und Materialverbrauchs (z. B. Abdosierung prüfen, Einsatz von Einwegprodukten minimieren, Möglichkeiten der Digitalisierung nutzen), Reduktion wenig gewinnbringender Prozesse.
7. Reduktion motorisierten Individualverkehrs, z. B. Mobilität durch Möglichkeiten der Digitalisierung nutzen (z. B. durch digitale Behandlungsoptionen, digitale Besprechungen/Konferenzen), um zur Reduktion der CO_2_-Emissionen und Schadstoffbelastung beizutragen.
8. Umsetzung einer vornehmlich pflanzenbasierten Verpflegung mit einem maximal geringen Anteil tierischer Produkte in Kliniken (Orientierung an Planetary Health Diet oder Empfehlungen der Deutschen Gesellschaft für Ernährung e. V.), da die pflanzenbasierte Ernährung dem Umweltschutz und der allgemeinen Gesundheit dienlich ist.
9. Berücksichtigung von Nachhaltigkeitskriterien bei der Beschaffung und Finanzierung (z. B. bei Anlageentscheidungen, im Einkauf oder bei der Forschungsfinanzierung).
10. Marketing für Nachhaltigkeit über Klinik- oder Praxiskommunikation (z. B. Klimasprechstunde).

### Infobox 3 Zum Positionspapier der DGPPN


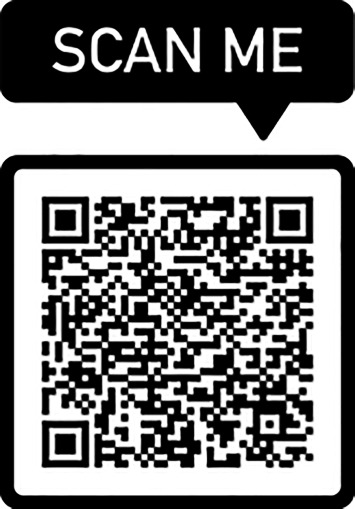


## Abkürzungen

BPM: Berufsverband der Fachärztinnen und Fachärzte für Psychosomatische Medizin und Psychotherapie Deutschlands
DGPM: Deutsche Gesellschaft für Psychosomatische Medizin und Ärztliche Psychotherapie
DGPPN: Deutsche Gesellschaft für Psychiatrie und Psychotherapie, Psychosomatik und Nervenheilkunde e. V.
DKPM: Deutsches Kollegium für Psychosomatische Medizin
IPCC: Intergovernmental Panel on Climate Change (Zwischenstaatlicher Ausschuss für Klimaänderungen, ugs. Weltklimarat)
PTBS: Posttraumatische Belastungsstörung
RCT: Randomisierte kontrollierte Studie
WHO: Weltgesundheitsorganisation
